# Association between dietary calcium to Phosphorus Ratio and the odds of ulcerative colitis: A case-control study

**DOI:** 10.1016/j.heliyon.2024.e27556

**Published:** 2024-03-06

**Authors:** Hadith Tangestani, Ali Jamshidi, Zahra Yari, Zahrasadat Jalaliyan, Hamid Ghalandari, Azita Hekmatdoost, Samaneh Rashvand, Amirhossein Mohammadi Baghmolae, Hadi Emamat

**Affiliations:** a Department of Nutrition, Bushehr University of Medical Sciences, Bushehr, Iran; b The Persian Gulf Tropical Medicine Research Center, The Persian Gulf Biomedical Sciences Research Institute, Bushehr University of Medical Sciences, Bushehr, Iran; c Department of Nutrition Research, National Nutrition and Food Technology Research Institute, Shahid Beheshti University of Medical Sciences Tehran, Tehran, Iran; d School of Medicine, Bushehr University of Medical Sciences, Bushehr, Iran; e Department of Community Nutrition, Shiraz University of Medical Sciences Shiraz University of Medical Sciences, Shiraz, Iran; f Department of Clinical Nutrition and Dietetics, National Nutrition and Food Technology Research Institute, Shahid Beheshti University of Medical Sciences, Tehran, Iran; g Students Research Committee, Bushehr University of Medical Sciences, Bushehr, Iran

**Keywords:** Inflammatory bowel diseases, Ulcerative colitis, Dietary intake, Dietary calcium, Dietary phosphorus

## Abstract

**Background & aims:**

Ulcerative colitis (UC) is a recurrent, inflammatory, autoimmune intestinal disease. The dietary calcium to phosphorus (Ca:P) ratio is suggested to affect the inividuals’ normal metabolic and inflammatory pathways. The present study aimed to investigate the association between dietary Ca:P ratio and the odds of developing UC in a case-control format.

**Methods:**

The study included sixty-two currently diagnosed UC patients and one hundred twenty-four matched controls, designed as a case-control study. The dietary intakes of the participants were assessed by a food frequency questionnaire (FFQ), and the dietary Ca:P ratio was calculated. The association between tertiles of Ca:P ratio and UC was examined using the logistic regression. P-values <0.05 were considered as significant.

**Results:**

The study sample consisted of participants with an average age of 36.63 ± 12.42 years and a mean body mass index (BMI) of 25.39 ± 3.82 kg/m^2^. The overall energy-adjusted ratio of Ca:P was 0.74 ± 0.11. In the multivariate model, after adjustment for potential confounders, participants in the third tertile of dietary Ca:P ratio had a lower odds of developing UC compared to the lowest tertlie (OR: 0.34, 95% CI: 0.13–0.87; p = 0.026).

**Conclusion:**

Our results indicate that a higher ratio of dietary Ca:P ratio might be protective against developing UC. However, further studies are warranted to examine this association in various populations.

## 1 Introduction

Inflammatory bowel diseases (IBDs) are recurrent autoimmune intestinal disorders. Crohn's disease (CD) and ulcerative colitis (UC) are prominent manifestations of IBDs in populations [1]. It has been postulated that a complex interactions of environmental, genetic, and immunoregulatory factors might be the origin of the chronic inflammation (i.e. the continuous presence of inflammatory cytokines in the bloodstream and tissues), which has been introduced as the major culprit in the pathogenesis of IBDs [1,2]. Common symptoms of UC include nausea, vomiting, lower left abdomen pain, diarrhea, constipation, weight loss, tenesmus, and rectal bleeding [3]. Studies have shown an increase of 31% in the worldwide occurrence of IBDs between the years 1990 and 2017, yielding a prevalence rate of 89.6 cases per 100,000 individuals [4]. In 2012, the annual incidences of IBDs and UC were reported to be 3.11 and 2.70 per 100,000 individuals in Iran, respectively. Moreover, the prevalence of IBDs and UC was reported as 40.67 and 35.52 per 100,000 individuals, respectively [5].

While the precise etiology of UC remains elusive, it is widely acknowledged as a multifactorial condition wherein genetics, immunity, and environmental factors collectively assume a crucial role in its pathogenesis [6]. Among the environmental factors, diet- and lifestyle-related elements are suggested to be effectual [7]. It has been noted that the intestinal microbiome, epithelial integrity, and mucosal immune system can be affected by dietary factors [8]. For instance, vitamins E and C, dietary antioxidants that protect against lipid oxidation and free radicals, may have a protective effect against developing UC, due to their ability to scavenge harmful oxygen and superoxide anion radicals [9]. A diet rich in dietary long-chain n-3 PUFAs may lower the risk of UC, while a higher dietary intake of *trans*-unsaturated fatty acids has been positively associated with its development. Additionally, several dietary habits (e.g. increased sugar and fat intake), reduced fiber consumption, insufficient intake of vitamins A and D have been postulated to impact the odds of UC and CD [10,11].

Likewise, dietary patterns characterized by excessive intake of refined sugar, fast foods, and red and processed meat have been associated with the development and recurrence of IBDs [12]. Fast foods and processed meats, which are often high in fat, are notable sources of inorganic phosphate, commonly used as a food additive to enhance taste, appearance, and shelf life [13,14]. It has been suggested that increased dietary phosphorus (P) intake may influence inflammatory diseases by altering cytokine levels; for example by increasing the concentration of interleukin-1β and decreasing the levels of interleukin-4 [15]. One animal study demonstrated that a high intake of dietary phosphorus in rats could be associated with exacerbated intestinal inflammation and increased expression of proinflammatory cytokines [13]. Furthermore, several experimental studies, as well as human trials, have reported the potential protective role of dietary calcium against intestinal infection with food-borne bacterial pathogens. Calcium (Ca) has been shown to exert some cytoprotective impacts in the colon through its involvement in the precipitation of bile acids and fatty acids, leading to declined cytotoxicity of the fecal stream. This process results in reduced intestinal epithelial cell damage and mucosal integrity enforcement [16]. A previous study showed that an above median Ca:P ratio in the diet could be negatively associated with central obesity measured by waist-to-height ratio (WHtR) [17]. Another study suggested that a low Ca:P ratio in the diet may have a negative impact on lipid metabolism; on the other hand, a positive association between the Ca:P ratio and serum HDL-cholesterol was observed [18].

In adults, dietary reference intakes (DRIs) for Ca and P are defined as 1200 mg/d and 700 mg/d, respectively; yielding an approximate Ca:P ratio of 1.7 (Ca:P), which is usually not met following the typical American dietary regimen [14]. Several studies have already investigated the association between either Ca or P with inflammatory disorders [13,15,19]. however, due to the increase in the consumption of processed foods containing high phosphorus additives and on the other hand, the decrease in the intake of calcium food sources in different communities and the adverse effects of decreasing the ratio of Ca:P on human health [14], this research was aimed to examine the relation of dietary ratio of Ca:P and the odds of UC in an Iranian population for the very first time.

## 2 Materials and methods

### 2.1 Subjects

The present case-control study was carried out at a referral hospital in Tabriz city, Iran, in 2013. The comprehensive details of the study protocol have already been reported [20]. Sixty-two currently diagnosed UC patients were included as the cases. The inclusion criteria for the cases was a recent UC diagnosis, determined via the relevant signs and symptoms, as well as certified colon tissue pathology reports. The exclusion criteria for the case group subject included a history of other gastrointestinal diseases, carcinoma, and other inflammatory, infectious, and autoimmune disorders. One hundred and twenty-four healthy subjects, matched in terms of age and sex, were included in the study as the control group.

Individuals without UC who were visiting orthopedic clinics within the same referral hospital were recruited to serve as the control group. The exclusion criteria in control group were having gastrointestinal illnesses/symptoms such as diarrhea, gastro-esophageal reflux disease (GERD), irritable bowel syndrome (IBS), and abdominal pains or any conditions that might have caused significant changes in the dietary habits. Finally, an age range of 20–80 years and not taking continuous vitamin and mineral supplements with anti-inflammatory or antioxidant effects were defined as other inclusion criteria for both the cases and the controls.

Participants were demanded to provide an informed consent before being enrolled in the study. Demographic information, medical history, body mass index (BMI), medication use, smoking status, *Helicobacter pylori* infection, education level, and family history of UC were collected. The study protocol received approval from the ethics committee of Bushehr University of Medical Sciences, with the ethical code: IR.BPUMS.REC.1402.027.

### 2.2 Dietary assessment

A valid and reliable FFQ was used by a trained interviewer to collect dietary intake data from participants over the past year [21]. The food composition tables provided by the U.S. departemtent of agriculture (USDA) were used to measure the daily intakes of Ca and P of the individuals. Moreover, calorie-adjusted amounts of the two minerals (the amount of each mineral in 1000 Kcals) and the Ca:P ratio were calculated.

### 2.3 Statistical analysis

The data was analyzed using SPSS software (version 21.0; SPSS, Chicago, IL, USA). Normality of the data was assessed using Kolmogorov–Smirnov test. The between-group comparisons regarding the qualitative and quantitative variables, were conducted using the chi-square and one-way ANOVA test, respectively. Subjects were divided into tertiles of energy-adjusted daily Ca:P ratio (the first tertile (T1): 0.39 to 0.7; the second tertile (T2): 0.71 to 0.8; the third tertile (T3): 0.8 to 1.04). A multiple logistic regression analysis was utilized to calculate the odds ratios (ORs) and 95% confidence intervals (CIs) of UC across tertiles. In all models, the first tertile was considered as the reference. In the final model, potential confounding variables, including age, sex, BMI, smoking, education, *Helicobacter pylori* infection, dietary intake of omega-3 fatty acids, *trans*-fatty acids, and total dietary fiber were adjusted. P-values less than 0.05 were considered as statistically significant.

## 3 Results

General characteristics of the participants across tertiles of the Ca:P ratio are represented in Table 1. The present study included 62 recently diagnosed patients with UC as cases and 124 healthy individuals as controls. Out of a total of 186 participants in the study, 81 were male, so that 27 were in the case group and 54 were in the control group. The participants' age range and body mass index (BMI) (mean ± SD) were 36.63 ± 12.42 years and 25.39 ± 3.82 kg/m^2^, respectively. There was no significant difference with regard to the general characteristics of participants across the tertiles of Ca:P ratio; except for BMI. Individuals in T3 had significantly higher BMI compared to the those in the first tertile (T1: 24.60, T2: 25.07, T3: 26.50 kg/m^2^; p = 0.01).

**Table 1:** Participants’ characteristics across tertiles of energy-adjusted dietary calcium to phosphorus ratio at the baseline.

	Tertiles of energy-adjusted dietary calcium to phosphorus ratio			
	T1 (n = 62)	T2 (n = 62)	T3 (n = 62)	p-value
Age (years)	34.75 ± 11.8	35.50 ± 13	39.64 ± 11.9	0.06
Male (%)	46.8	41.9	41.9	0.82
BMI (kg/m^2^)	24.60 ± 3.9	25.07 ± 3.3	26.50 ± 3.9	0.01
Education (%)				0.14
• Primary	6.5	1.6	12.9	
• Secondary and high school	50	53.2	53.2	
• University	43.5	45.2	33.9	
Smoking (%)	9.7	9.7	6.5	0.76
H. pylori infection (%)	6.5	1.6	4.8	0.40
Family history of UC (%)	3.2	0	0	0.13

Data are presented as mean (± standard deviation (SD)) for continuous variables and percent for categorically distributed variables.

BMI: Body mass index, UC: Ulcerative colitis.

The dietary intakes of participants across tertiles of the energy-adjusted Ca:P ratios are reported in Table 2. Individuals in the first tertile had a relatively higher intake of total fat, monounsaturated fatty acid (MUFA), and polyunsaturated fatty acid (PUFA) than the T2 and T3 (p < 0.01), while the mean intake of dietary fiber was the lowest (p < 0.001). The dietary Ca intake among all participants was 1160 ± 359 mg/day (mean ± SD). The dietary P intake among all participants was 1560.41 ± 424.61 mg/day (mean ± SD), with no significant differences among the tertiles. The overall energy-adjusted ratio of Ca:P was 0.74 ± 0.11 (mean ± SD) (range: 0.39–1.01).

**Table 2:** Dietary intakes of participants across tertiles of energy-adjusted dietary calcium to phosphorus ratio.

Tertiles of energy-adjusted daily calcium to phosphorus ratio intake				
	T1 (n = 62)	T2 (n = 62)	T3 (n = 62)	p-value
Total calories (kcal/day)	2797.7 ± 673.7	2680.2 ± 578.1	2605.9 ± 602.4	0.22
Total carbohydrate (g/day)	367.1 ± 97.1	358.2 ± 73.8	362.7 ± 93.1	0.85
Total fat (g/day)	110.5 ± 29.5	103.1 ± 32.3	94.1 ± 25.7	0.009
Total protein (g/day)	97.90 ± 32.24	92.77 ± 26.37	87.10 ± 23.87	0.09
SAFAs (g/day)	30.85 ± 10.08	29.91 ± 11.65	28.53 ± 10.38	0.47
MUFAs (g/day)	38.21 ± 10.96	34.35 ± 10.51	31.48 ± 8.76	0.001
PUFAs (g/day)	26.48 ± 8.26	24.28 ± 7.88	21.28 ± 6.72	0.001
*Trans*-fatty acids (g/day)	0.027 ± 0.066	0.019 ± 0.076	0.027 ± 0.088	0.81
Total dietary fiber (g/1000 kcal)	17.91 ± 5.99	20.27 ± 6.27	25.74 ± 8.29	<0.001
Calcium (mg/day)	969.35 ± 310.47	1175.77 ± 324.34	1335.66 ± 347.43	<0.001
Phosphorus (mg/day)	1577.45 ± 446.55	1558.08 ± 415.99	1545.71 ± 416.92	0.91

Data are presented as mean ± standard deviation.

PUFA: polyunsaturated fatty acid; MUFA: monounsaturated fatty acid; SAFA: saturated fatty acid.

The odds ratio (95% CI) of UC across energy-adjusted tertiles of daily Ca:P ratio are presented in Table 3. In the crude model, individuals with the highest Ca:P intake (T3) had 58% lower odds of being inflicted with UC compared with those in the lowest tertile (OR: 0.42, 95% CI: 0.19–0.91; p = 0.026). In the second model, after adjusting for age and sex, the negative relationship remained meaningful (OR: 0.38, 95% CI: 0.17–0.84; p = 0.017). After additional adjustments in final model, not only the inverse association remained significant, it was further highlighted (OR: 0.34, 95% CI: 0.13–0.87; p = 0.026).

**Table 3:** The OR (95%CI) of UC across tertiles of energy-adjusted dietary calcium to phosphorus ratio.

Tertiles of energy-adjusted daily calcium to phosphorus ratio intake					
	T1 (n = 62)	T2 (n = 62)	P-Value	T3 (n = 62)	P-Value
Cases/controls	28/34	18/44		16/46	
Range of calcium to phosphorus ratio	0.39 to 0.7	0.71 to 0.8		0.8 to 1.04	
aModel 1	1.00 (Ref)	0.49 (0.23–1.04)	0.06	**0.42 (0.19**–**0.91)**	**0.026**
bModel 2	1.00 (Ref)	0.48 (0.23–1.02)	0.06	**0.38 (0.17**–**0.84)**	**0.017**
cModel 3	1.00 (Ref)	0.52 (0.23–1.17)	0.11	**0.34 (0.13**–**0.87)**	**0.026**

a Crude model.

b Adjusted for age and gender.

c Additionally adjusted for BMI, smoking, education, Helicobacter pylori infection, dietary omega-3 fatty acids, *trans*-fatty acids, and total dietary fiber.

## 4 Discussion

To our understanding, we believe that our study represents the first investigation to explore the association between Ca:P ratio and the odds of UC in a case-control framework. We observed that the lower ratio of Ca:P was associated with an increased odds of UC.

Although previous studies have shown the role of various nutrients in the initiation, exacerbation, or prevention of intestinal inflammation [22], the studies which have considered Ca:P ratio as the main factor seem to be scarce in the literature. However, the protective effects of calcium and the deleterious effects of phosphorus in the pathogenesis of IBDs have been observed in experimental models [13,19]. The effects of dietary calcium in improving intestinal permeability and reducing diarrhea, one as a known etiologic precursor and one as a common symptom of IBDs, have been previously demonstrated. Since increased permeability is usually attributed to the relapse of the IBDs, the beneficial effect of calcium on intestinal epithelial integrity (i.e. reduced permeability) may be of great significance [23]. Furthermore, dietary calcium appears to provide partial protection against inflammation by maintaining intestinal barrier function.

In an experimental model of IBDs, Schepens et al. have indicated the possible protective effect of Ca on colitis [16]. In this study, human leukocyte antigen (HLA)-B27 transgenic mice were fed with pure high-fat diet consisting of low or high amounts of calcium. They observed that mucosal IL-1β levels (as a potent pro-inflammatory marker) and histological colitis scores were significantly lower in calcium-fed mice. The results of the histological assessment indicate that the administration of calcium had a preventive effect on the colitis-induced increase in the expression of extracellular matrix remodeling genes [16].

Some other mechanisms have been reported to explain the impact of impaired Ca metabolism on the inflammatory state which is a prominent feature of IBDs. For instance, loss of calcium-sensing receptor (CaSR) in the intestinal epithelium, a dimeric G protein-coupled receptor (GPCR) which has a crucial function in maintaining calcium homeostasis, compromises the epithelial barrier, fascilitating the penetration of pathogens which could subsequently flare up the pro-inflammatory pathways in the body [24]. In line with these observations, the inverse relationship between high calcium intake and reduced risk of intestinal inflammation and colorectal cancer has been previously uncovered [25]. It has also been postulated that calcium has the potential to reduce the cytotoxicity of intestinal contents by forming insoluble complexes with phosphate-containg compounds in the upper part of the small intestine. These complexes could eventually bind to ductal bile acids and fatty acids. Through this mechanism, the delicate colon epithelial might be protected from some irritants which are considered partially responsible for the inflammatory outburst obsereved in IBDs [26].

Furthermore, the formation of Ca–P complexes reduce levels of free phosphorus, which in turn exerts further amelioration in the inflammatory state of the body. There has been some evidence with respect to the increased blood levels of phosphorus and the concentration of pro-inflammatory cytokines, including IL-1β [15]. In addition, epidemiologic investigations have reported that the consumption of high-P foods (such as fast foods and processed meats) might be accountable in the increased risk of IBDs onset and recurrence [27,28]. Dietary P has also been observed to increase intestinal inflammation in a dose-dependent manner, possibly through activation of nuclear factor-kappa B (NF-kB) (a factor responsible for the transcription of numerous pro-inflammatory activities), induction of proinflammatory cytokines, and an augmented recruitment of macrophages [23]. Along with inflammation, oxidative stress has also been associated with the pathogenesis of IBDs [29]. High P loading has been shown to directly increase mitochondrial generation of reactive oxygen species (ROSs), which has been linked to the calcification of intestinal endothelium and, subsequently, endothelial dysfunction [30].

An experimental study showed that the increase in phosphorus load in intestinal cells leads to the stimulation of inflammatory pathways, the increase in the production of reactive oxygen species (ROS), the activation of macrophages, and ultimately colitis damages [13]. Nonetheless, adverse effects of excess phosphorus are rarely observed and may occur with either superfluous intake of dietary phosphate intake or a deficiency of dietary calcium. If the ratio of calcium to phosphorus is balanced, i.e. a ratio of about 2:1, a wide range of phosphorus levels will be tolerated [31].

The currect study has some strengths. Firstly, to the best of our knowledge, this investigation is unprecendented in examining the association between of Ca:P ratio and the risk of UC in a case-control format. Secondly, enrollment of newly diagnosed subjects might have reduced the impact of information bias, more specifically recall bias. Thirdly, to avoid interviewer bias, only one interviewer was employed to solicit the relevant data from the participants. Nonetheless, the present research had some weakness. Firstly, owing to the observational nature of the case-control design, drawing a causal relationship was rendered impossible. Secondly, despite the efforts made to prevent it, since the questionnaires which were used depended greatly on the individuals’ recalling capacity, information bias cannot be thoroughly ruled out. It is also suggested that serum inflammatory biomarkers be investigated in future studies to confirm our results.

## 5 Conclusion

The findings of present study demonstrate an inverse association between the dietary Ca:P ratio and the odds of UC. These observations are in line with those of previous investigations. However, due to the observational nature of this research, further studies, especially in the format of human clinical trials, are needed to derive an empirical causal association.

## Ethics approval and consent to participate

The study was approved by the Ethics Committee of Bushehr University of Medical Sciences. The ethical committee code was IR.BPUMS.REC.1402.027. Written informed consent was obtained from all participants.

## Data availability statement

The datasets generated and/or analyzed during the current study will be available from the corresponding author on reasonable request.

## Funding

We appreciate the “Student Research Committee” and “the Persian Gulf Martyrs Hospital's Clinical Research Development Center” in 10.13039/501100002769Bushehr University of Medical Sciences for their financial support of this study.

## CRediT authorship contribution statement

**Hadith Tangestani:** Writing – review & editing, Writing – original draft. **Ali Jamshidi:** Writing – original draft. **Zahra Yari:** Writing – original draft. **Zahrasadat Jalaliyan:** Writing – review & editing, Supervision. **Hamid Ghalandari:** Writing – review & editing. **Azita Hekmatdoost:** Project administration, Methodology, Data curation, Conceptualization. **Samaneh Rashvand:** Methodology, Data curation, Conceptualization. **Amirhossein Mohammadi Baghmolae:** Writing – original draft. **Hadi Emamat:** Writing – review & editing, Supervision, Funding acquisition, Formal analysis, Conceptualization.

## Declaration of competing interest

The authors declare that they have no known competing financial interests or personal relationships that could have appeared to influence the work reported in this paper.
